# From Digital Planning to Surgical Precision: Assessing the Accuracy of NemoFAB in Orthognathic Surgery

**DOI:** 10.3390/jcm15020532

**Published:** 2026-01-09

**Authors:** Robert-Paul Avrămuț, Serban Talpos, Andra-Alexandra Stăncioiu, Alexandru Cătălin Motofelea, Malina Popa, Camelia-Alexandrina Szuhanek

**Affiliations:** 1 Orthodontic Research Center ORTHO-CENTER, Discipline of Orthodontics I, Faculty of Dental Medicine, “Victor Babes” University of Medicine and Pharmacy Timisoara, 9 No., Revolutiei Bv., 300041 Timisoara, Romania; robert.avramut@umft.ro (R.-P.A.); cameliaszuhanek@umft.ro (C.-A.S.); 2 Discipline of Oral and Maxillo-Facial Surgery, Faculty of Dental Medicine, “Victor Babes” University of Medicine and Pharmacy Timisoara, Revolutiei Boulevard 9, 300041 Timisoara, Romania; 3 Center for Molecular Research in Nephrology and Vascular Disease, Discipline of Nephrology, Department VII/Internal Medicine II, Faculty of Medicine, “Victor Babes” University of Medicine and Pharmacy, 300041 Timisoara, Romania; alexandru.motofelea@umft.ro; 4 Department of Pediatric Dentistry, Faculty of Dental Medicine, “Victor Babes” University of Medicine and Pharmacy, Eftimie Murgu Square 2, 300041 Timisoara, Romania; popa.malina@umft.ro

**Keywords:** CBCT imaging, dento-maxillary deformities, digital splint fabrication, interdisciplinary treatment, maxillofacial surgery, orthodontic decompensation, orthodontic–surgical approach, orthognathic surgery, treatment workflow, virtual surgical planning

## Abstract

**Background/Objectives**: Three-dimensional virtual surgical planning (VSP) is increasingly central to contemporary orthognathic surgery, enhancing diagnostic precision and enabling more reliable forecasts of postoperative outcomes. NemoFAB (Nemotec, Madrid, Spain) is a recently developed digital platform that integrates CBCT data, digital dental models, and facial photographs into a single workflow. Despite its growing clinical use, independent validation of its predictive accuracy remains limited. This study evaluated how closely NemoFAB virtual predictions corresponded to actual postoperative results using standardized cephalometric parameters. **Methods**: Forty adult patients with dento-maxillofacial deformities requiring combined orthodontic–surgical treatment were included. Eleven cephalometric variables—common to both WebCeph (2D) and NemoFAB (3D)—were measured preoperatively, virtually in NemoFAB, and postoperatively. AI-assisted landmark placement was manually verified by two orthodontists. Statistical analyses included repeated-measures ANOVA, paired *t*-tests, Bland–Altman plots, and intraclass correlation coefficients (ICC) to evaluate agreement and predictive accuracy. **Results**: Overjet, overbite, maxillary incisor inclination, maxillary incisor exposure, mandibular incisor projection to the True Vertical Line, and occlusal plane angulation all showed statistically significant changes after surgery (*p* < 0.05). Bland–Altman analysis demonstrated the narrowest limits of agreement in Nemo–Post comparisons, indicating strong predictive alignment. ICC values showed excellent agreement for incisor angulation (ICC = 0.921–0.984) and Pogonion projection (ICC = 0.943). Consistently poor pre-Nemo agreement reflected expected discrepancies between initial anatomy and planned surgical correction. **Conclusions**: When predicting skeletal and dentoalveolar changes brought about by orthognathic surgery, NemoFAB showed a high degree of agreement, especially for parameters that are directly impacted by jaw repositioning. Its strong concordance with postoperative outcomes supports its reliability for virtual planning, interdisciplinary coordination, and surgical execution. Advances in soft-tissue modeling and AI-driven automation may further enhance predictive accuracy.

## 1. Introduction

The formulation of an accurate pre-surgical strategy is crucial in the surgical management of dentofacial abnormalities. Two-dimensional (2D) radiographs and manual model surgery are critical components of preoperative preparation for orthognathic surgery. This method has drawbacks, particularly for individuals with significant facial deformities or asymmetry, since 2D cephalometric scans fail to provide comprehensive information about 3D features. During the implementation of traditional 2D surgical designs, unforeseen complications may arise, including bone collisions in the ramus region, discrepancies in pitch, roll, and yaw rotation, midline deviations, and chin deficiencies [1].

During two-jaw surgery, an inter-occlusal splint is constructed to serve as an intermediary guide for the repositioning of the maxilla in relation to the intact mandible. Any discrepancy between the surgical plan and the plaster model may result in a badly constructed wafer, subsequently leading to unforeseen and frequently unfavorable outcomes, irrespective of the surgeon’s expertise and diligence [2]. These examples demonstrate that the formulation of a detailed pre-surgical strategy is crucial for rectifying dentofacial abnormalities [1].

Computer-aided surgical simulations using cone beam computed tomography (CBCT) images have transformed orthodontics and have been applied for orthognathic surgery (OGS) to enhance cephalometric analysis, surgical simulation, and splint production [3,4,5,6,7,8].

The visualization of skeletal complexities in asymmetric dentofacial deformities has significantly improved through three-dimensional (3D) modeling, which illustrates the degree of yaw rotation in the maxilla and mandible, occlusal plane inclination, and variations in the length of the mandibular body or ramus [9,10,11].

The 3D simulation technique has been used for planning in orthognathic surgery, resulting in substantial improvements in surgical results [1]. Intraoperative efficiency has improved due to the creation of templates and jigs that replicate the gaps or spacing between the osteotomies shown in the virtual design. These jigs may enhance intraoperative precision of the clinical execution of the virtual plan and assist in the orientation and placement of osseous segments [1].

The use of digital technology in orthognathic surgery is one of the most significant innovations in maxillofacial surgery since the introduction of rigid fixation, as shown by the plethora of published studies on the topic. The use of digital technology has enhanced diagnostic precision, treatment planning, and conventional therapeutic results from both functional and aesthetic perspectives [12,13].

Orthognathic surgery is a meticulous procedure that depends on thorough preoperative planning, precise execution of the chosen surgical plan, and diligent postoperative care for success. Over the last sixty years, orthognathic surgery has progressed to be safer, more efficient, less expensive, and more effective. Conventional surgical planning (CSP), including manual model surgery, photographic analysis, and two-dimensional radiography, has traditionally been used during the preoperative phase of orthognathic surgery. Virtual surgical planning (VSP) methodologies have progressively been used as a substitute for traditional surgical planning (TSP). The computer-assisted surgical simulations used in VSP provide surgeons with a three-dimensional (3D) model of the facial bones, soft tissue, and dentition to enhance virtual diagnosis and surgery [14].

For the sake of clarity, computer-assisted surgical simulation workflows are referred to throughout the manuscript as Virtual Surgical Planning (VSP).

The comparison between VSP and TSP is complex across several outcomes. Although VSP has been portrayed as a better alternative to TSP, academics have raised doubts on whether the accuracy of the VSP approach surpasses that of TSP [15].

TSP has been recognized as better in terms of cost efficiency. Nonetheless, the TSP cost benefit emerged subsequent to the first fixed cost investment in VSP. In their systematic review and meta-analysis, Chen et al. determined that VSP and TSP have equivalent predictive accuracy in the sagittal plane. The results underscore the need to assess particular outcomes of VSP and TSP in the context of a given osteotomy [16].

Bimaxillary osteotomy is a prevalent yet intricate surgical intervention requiring meticulous planning and precise execution. Accuracy, duration, and expense are critical factors when assessing the treatment protocol in bimaxillary osteotomy. The duration to be evaluated when comparing the efficacy of VSP and TSP includes planning time, operational time, and overall working/doctor time. Shorter surgical timeframes are preferable since they mitigate risk and reduce anesthesia and blood loss, while abbreviated planning time prevents delays in required or urgent treatment. The overall duration aids in assessing the effectiveness and expense of the methodology used in bimaxillary osteotomy [14].

Orthognathic surgery, a subset of oral and maxillofacial surgery, corrects dentofacial abnormalities in individuals with diverse malocclusions. The main objectives of orthognathic surgery are to augment mastication, respiration, and phonation, as well as to enhance face aesthetics. Successful orthognathic surgery requires meticulous collaboration between the surgeon and orthodontist, with the orthodontist overseeing pre- and postoperative orthodontics to achieve optimal occlusion for both functional and aesthetic results. Precise treatment planning is essential for effective orthognathic surgery. A comprehensive diagnostic strategy is crucial for simulating surgical operations and forecasting orthodontic motions, hence, achieving accurate occlusion. Treatment planning and presurgical procedures, including the tooth–jaw connection, osteotomy location, and surgical method, are essential for favorable surgical results. Traditional surgical planning (TSP) for orthognathic surgery includes clinical and physical assessments, photographic documentation, two-dimensional cephalometric radiographs, model analysis, and the use of a face bow with a semi-adjustable articulator. Nonetheless, the several processes inherent in TSP may result in cumulative mistakes and are labor-intensive [17].

Recent innovations, including cone-beam computed tomography (CBCT) scanning and three-dimensional surface technology integrated with computer-aided design software, have transformed orthognathic surgery. These technologies enable virtual orthognathic planning (VOP) via the acquisition of CBCT data and the analysis of digital dental models. The alignment of the dental arch and individual teeth may be assessed in relation to the patient’s face and cranial structure. Moreover, three-dimensional data, cephalometric measurements, and virtual surgical planning improve the accuracy of predicting the relationship between movements of the dentoskeletal complex and soft tissue reaction. Virtual surgical planning (VSP) involves many stages, including data acquisition, pre-planning consultations, strategic planning, and the fabrication of surgical splints to guarantee precise diagnosis and treatment [17].

Recent research has emphasized the significance of virtual 3D prediction in attaining favorable outcomes in orthognathic surgery. Stokbro et al. evaluated five clinical cases, indicating that the VSP technique showed significant predictability and consistency for jaw surgery [18].

Nonetheless, the lack of clinical control rendered it difficult to ascertain whether the planning technique was preferable. Gaber et al. performed a comprehensive study to assess the precision of VSP and developed a uniform methodology with a small error margin, including automated or semi-automated assessment, inter- and intra-observer reliability, and voxel registration at the cranial base [19].

Researchers, however, challenge the accuracy of the existing VSP approach in comparison to the TSP technique [20].

Since 2003, several studies have evaluated the accuracy, duration, expense, and patient satisfaction with TSP and VSP [21].

A recent comprehensive study by Nilsson et al. has shown that, in contrast to conventional techniques, computer-assisted design reduced surgical and ischemic durations for maxillofacial reconstruction and abbreviated preoperative planning periods for orthognathic surgery [22].

In this context, NemoFAB (Nemotec, Madrid, Spain) represents an advanced digital platform designed for comprehensive orthodontic and orthognathic planning [16,20,21,23]. The software integrates three-dimensional cone-beam computed tomography (CBCT) data, digital dental models, and facial photographs to enable accurate virtual diagnosis, cephalometric analysis, and surgical simulation within a single interface [6,11,12]. By combining orthodontic decompensation, virtual osteotomies, and occlusal adjustments, NemoFAB provides a continuous workflow from digital prediction to surgical execution. Its simulation modules allow for visualization of skeletal movements and postoperative occlusal relationships, facilitating interdisciplinary communication between orthodontists and surgeons. Despite its growing adoption in clinical practice, independent studies evaluating the predictive accuracy and clinical reliability of NemoFAB remain limited, highlighting the need for objective validation of its virtual planning outcomes against actual postoperative results [1,13,19].

Over the years, several soft tissue studies were created, placing increasing focus on the clinical investigation of soft tissue function and aesthetics [24,25,26,27,28]. Nonetheless, the reference planes used were either the Frankfort horizontal plane or the cranial base, whose orientation may vary considerably across individuals [29]. To address these deficiencies, Arnett et al. [30] devised a novel soft-tissue cephalometric analysis (STCA) grounded on natural head position (NHP) and a true vertical line (TVL) as the reference plane for assessing soft tissue norms. It soon became evident that establishing standards for multiple races was necessary due to clinically substantial variances in craniofacial morphology and soft tissues across diverse ethnic groups, since normative data from one group does not accurately reflect others. The notion of floating norms arose to tailor treatment criteria based on distinct dental and skeletal patterns. The evaluation of growth-related changes in soft-tissue profile characteristics may be essential for effective treatment planning and forecasting the stability of outcomes [29].

The referenced literature includes studies that specifically assess the precision of virtual surgical planning in orthognathic surgery as well as more general studies that discuss the advancements in digital imaging, cephalometric frameworks, and computer-assisted workflows that have influenced the creation of modern planning systems. The referenced studies offer crucial methodological and conceptual context for comprehending the justification, validation techniques, and clinical integration of contemporary virtual planning technologies, even though not all of them explicitly evaluate orthognathic surgical accuracy.

The primary aim of this study was to evaluate the accuracy and reliability of the NemoFAB virtual surgical planning software in predicting postoperative skeletal and dental outcomes in patients undergoing orthognathic surgery. Specifically, the research sought to determine the degree of concordance between virtual surgical simulations and actual postoperative results, as measured through standardized cephalometric analyses.

Secondary objectives included assessing the reproducibility of cephalometric landmark identification using AI-assisted tools (WebCeph) and verifying the clinical applicability of digital prediction workflows in achieving precise and clinically acceptable orthognathic outcomes.

## 2. Materials and Methods

In the course of the research, the focus was placed on patients who were enrolled in the Orthodontics I Discipline at the Faculty of Dental Medicine at the “Victor Babes” University of Medicine and Pharmacy Timisoara. The Institutional Ethics Committee of the “Victor Babes” University of Medicine and Pharmacy Timisoara, Romania, approved the research, and all participants provided their written informed consent for the study (CECS Nr. 105/02.11.2022 rev 2025).

The study included patients who underwent orthognathic surgery between November 2022 and November 2024, with a minimum postoperative follow-up period of six months.

At the Faculty of Dental Medicine, “Victor Babes” University of Medicine and Pharmacy, Timisoara, forty adult patients from Romania (adults with a median age of 29.0 years (IQR: 26.0–34.0), with 19 (48%) females and 21 (53%) males) underwent combined orthodontic and orthognathic treatment.

The study cohort reflected the typical orthognathic surgery population treated in university—predominantly young adults motivated by both functional and esthetic considerations. Before virtual surgical planning and the following orthognathic correction, each patient had finished or was in the process of finishing presurgical orthodontic decompensation. Comprehensive diagnostic documentation was available for all participants, including panoramic radiographs (OPG), lateral and frontal cephalometric radiographs, cone-beam computed tomography (CBCT) scans, standardized extraoral and intraoral photographs, and digital study models acquired using an intraoral scanner (3Shape Trios). This comprehensive clinical and radiographic dataset facilitated precise preoperative evaluation, virtual surgical planning and three-dimensional cephalometric analyses were performed using the NemoFAB software (Nemotec, Madrid, Spain), part of the NemoStudio suite (version 25.0.0.0; May 2025).

All patients were diagnosed with dento-maxillary deformities requiring combined orthodontic–surgical treatment.

Preoperative and postoperative records were collected for all subjects, ensuring a minimum follow-up period of six months after surgery. The collected data were analyzed to assess skeletal and dental changes and to evaluate the accuracy of virtual surgical planning with NemoFAB compared to the actual postoperative outcomes.

Study Population

A total of forty Romanian patients who presented to the university orthodontic center seeking improvement in facial aesthetics, dental alignment, and functional occlusion were included in this study.

Each participant underwent a comprehensive diagnostic assessment, which included panoramic radiographs (OPG), lateral cephalograms, intraoral and extraoral clinical photographs, and digital dental models obtained through intraoral scanning.

All patients were diagnosed with dento-maxillofacial deformities requiring combined orthodontic–surgical management and were treated within the interdisciplinary collaboration between the Department of Orthodontics I and Maxillofacial Surgery.

Inclusion and Exclusion Criteria

Patients were considered eligible for orthognathic surgery if they met all of the following criteria:

- Were at least 18 years of age at the time of the initial evaluation;
- Presented with a dento-maxillofacial deformity requiring surgical correction, including but not limited to skeletal Class II or Class III malocclusion, vertical maxillary excess or deficiency, or transverse discrepancies not manageable with orthodontic treatment alone;
- Had completed or were in the process of completing presurgical orthodontic decompensation;
- Were in good general health (ASA I or II) and had no systemic disease contraindicating general anesthesia;
- Possessed complete diagnostic documentation, including CBCT scans, cephalometric radiographs, intraoral scans, and standardized clinical photographs;
- Provided written informed consent for treatment, data analysis, and follow-up assessments.

Exclusion criteria comprised

- Patients under 18 years of age;
- Presence of craniofacial syndromes or congenital anomalies (e.g., cleft lip and palate, hemifacial microsomia);
- Temporomandibular joint disorders requiring separate or adjunctive surgical intervention;
- Poor oral hygiene status or active periodontal disease at the time of surgical planning;
- severe psychiatric or behavioral disorders compromising treatment adherence or Postoperative follow-up;
- Incomplete diagnostic records or refusal to undergo orthodontic treatment;
- and lack of written informed consent.

### 2.1. Procedure Methodology

Cephalometric Image Acquisition and Processing

All data handling procedures adhered to the General Data Protection Regulation (GDPR) and to the compliance standards of the digital platform used.

After creating the patient profile within the WebCeph environment, each lateral cephalometric radiograph was uploaded to the platform’s dedicated cephalometric analysis module.

The AI-based Landmark Detection tool automatically identified the key anatomical reference points on each image, after which AI-assisted digitization was applied to convert the radiograph into a quantitative, measurable format.

To ensure spatial precision, image calibration was conducted using the integrated scaling tool. A reference length of 10 mm was established on each radiograph, resulting in a pixel resolution of 4294 px/mm. This calibration process ensured consistent dimensional accuracy across different imaging systems.

High image quality was maintained by optimizing technical parameters: image size 3000 × 2400 pixels, resolution 300 dpi, and 24-bit color depth.

These specifications guaranteed optimal edge definition and grayscale differentiation, enabling both the artificial intelligence system and the human examiner to reliably identify anatomical landmarks.

By maintaining sufficient spatial resolution, we minimized scaling and calibration errors between patients and imaging sessions, thereby supporting reproducible cephalometric measurements.

Cephalometric Landmark Verification and Parameter Extraction

After automatic detection of anatomical landmarks by the WebCeph artificial intelligence module, all points were carefully inspected and, when necessary, manually adjusted to ensure anatomical precision. Two experienced orthodontists independently reviewed every tracing, and discrepancies were resolved by consensus to guarantee inter-examiner reliability.

Once verified, the finalized landmark configuration was stored within the software, completing the preprocessing stage prior to analysis.

Cephalometric measurements were then generated automatically using the WebCeph Analysis (version 2.0.0; AssembleCircle Corp., Seongnam-si, Gyeonggi-do, Republic of Korea). The software computed a full set of skeletal and dental parameters, from which only the variables predefined in the study design were selected for statistical evaluation.

Both preoperative and postoperative radiographs were analyzed following the same standardized protocol to ensure methodological consistency.

The combined use of validated AI-based software, expert landmark verification, and standardized imaging procedures contributed to high measurement reproducibility and minimized operator-dependent bias throughout the investigation.

Cephalometric analyses were performed using the WebCeph software platform (AssembleCircle Corp., Pangyoyeok-ro, Bundang-gu, Seongnam-si, Gyeonggi-do, Republic of Korea), a cloud-based digital tracing system officially registered with the Korean Intellectual Property Office (Seoul, Republic of Korea).

This platform uses advanced AI algorithms to automatically find and analyze cephalometric landmarks. Its proprietary technologies are protected by patents issued by both the Korean and United States Intellectual Property Offices, confirming the system’s technological reliability and innovation in AI-assisted cephalometric evaluation.

Standard Cephalometric Parameters Utilized in WebCeph and NemoFAB.

Common Cephalometric Parameters—For the purposes of this study, the following cephalometric parameters were evaluated using both WebCeph (2D platform) and NemoFAB (3D virtual planning software). These parameters were selected to ensure consistent measurement of dental, skeletal, and soft-tissue changes and to facilitate direct comparison between pre-operative planning and post-operative results.

Three-dimensional cephalometric analysis was performed using cone-beam computed tomography (CBCT) data and digital dental models integrated in NemoFAB (Nemotec^®^, Madrid, Spain). All subjects were scanned in natural head position (NHP) with the mandible in maximum intercuspation. DICOM data were imported into NemoFAB and registered to maxillary and mandibular STL models using surface-based alignment methods. The analysis followed the soft-tissue cephalometric principles described by Arnett and Gunson [30,31].

Reference Planes and Coordinate Systems

A standardized reference system was constructed for all measurements.

The True Vertical Line (TVL) was drawn through the subnasale (Sn) with the patient positioned in NHP. This landmark-based TVL was chosen according to the original Arnett–Gunson soft-tissue cephalometric analysis [30,31] and its later validation for orthognathic surgery planning by Espinar-Escalona et al. [32].

The maxillary occlusal plane (MxOP) was defined using the mesiobuccal cusps of the maxillary first molars and the midpoint of the maxillary central incisors.

Similarly, the mandibular occlusal plane (MdOP) was defined using the corresponding mandibular landmarks.

All planes and coordinate axes were generated automatically by NemoFAB following manufacturer guidelines and validated CBCT workflows [33,34].

Cephalometric Landmarks and Measurements

AI-assisted detection was used to first identify cephalometric landmarks, which were then manually verified and adjusted. Each measurement was taken twice, and the average value was used for analysis. Eleven parameters were evaluated, classified as dental relations, incisor inclination, vertical maxillary parameters, and TVL-based sagittal measures.

Dental Relationship Parameters

1. Overjet (mm)

Horizontal distance between the labial surface of the maxillary central incisor (Mx1) and the corresponding mandibular central incisor (Md1), measured parallel to the occlusal plane [30].

2. Overbite (mm)

Vertical overlap of the maxillary incisor edge over the mandibular incisor edge, measured perpendicular to the occlusal plane [30].

Incisor Inclination Parameters

3. Mx1 to Maxillary Occlusal Plane Angle (°)

Angle between the long axis of the maxillary central incisor and the maxillary occlusal plane (MxOP), expressing maxillary incisor inclination.

4. Md1 to Mandibular Occlusal Plane Angle (°)

Angle between the long axis of the mandibular central incisor and the mandibular occlusal plane (MdOP), reflecting lower incisor inclination.

Vertical Maxillary Parameters

5. Maxillary Incisor Exposure (mm)

Vertical distance from stomion superius to the incisal edge of the maxillary central incisor at rest. Defined according to soft-tissue cephalometric principles described by Arnett and Gunson [30,31].

6. Maxillary Anterior Height (mm)

Linear distance from the subnasale (Sn) to the incisal edge of Mx1, representing the vertical position of the anterior maxilla [30].

TVL-Based Sagittal Parameters (Arnett–Gunson)

All sagittal measurements were obtained as linear horizontal projections to the True Vertical Line (TVL), as described in Arnett’s original STCA system [30,31] and applied in 3D facial analysis [32].

7. Mx1 to TVL (mm)

Horizontal projection of the maxillary central incisor onto the TVL, indicating sagittal maxillary incisor position.

8. Md1 to TVL (mm)

Horizontal projection of the mandibular central incisor to TVL, assessing sagittal mandibular incisor position.

9. B-Point to TVL (mm)

Horizontal distance of skeletal point B from the TVL, representing sagittal mandibular basal position.

10. Pogonion to TVL (mm)

Horizontal distance from the Pogonion (hard-tissue Pog) to the TVL, used to evaluate chin prominence [32].

11. Maxillary Occlusal Plane to TVL (mm)

A horizontal line is drawn from a representative spot on the upper jaw’s biting surface to the True Vertical Line (TVL). This measurement assesses the forward or backward location of the maxilla’s dental base in the sagittal plane. In both NemoFAB and WebCeph, this measurement was recorded in millimeters (mm) as a linear projection of the TVL.

Study results were predefined as primary and secondary variables for clinical interpretation. The main results included cephalometric parameters (overjet, overbite, maxillary incisor inclination, maxillary incisor exposure, mandibular incisor projection to the true vertical line (TVL), and maxillary occlusal plane orientation) that directly reflected sagittal and vertical dentoskeletal changes attained through orthognathic surgery and targeted by virtual surgical planning. Secondary outcomes included parameters such as mandibular incisor inclination, maxillary height, B-point projection, and pogonion projection to the TVL that represented biologically stable skeletal landmarks or supportive dental relationships.

Software Measurement Protocol

All measurements were calculated using NemoFAB’s built-in 3D cephalometric tools. The software automatically generated reference planes and projections after landmark placement. Measurement techniques and 3D superimpositions followed validated CBCT methods described by Cevidanes et al. [33] and 3D orthodontic imaging standards described by Hajeer et al. [34].

Lateral cephalometric radiographs were collected preoperatively and postoperatively utilizing similar acquisition techniques to guarantee methodological consistency and measurement reliability. All pictures were obtained using the same radiography device, which was calibrated before each session to ensure consistent output parameters, including focal distance, tube voltage, tube current, and exposure length. A single qualified radiology technician executed each acquisition in accordance with a consistent operating method, thereby reducing operator-related variability.

Patients were positioned in a consistent natural head posture (NHP), ensuring central occlusion and a relaxed lip position. The Frankfort horizontal plane was meticulously oriented parallel to the floor, and this alignment was confirmed using the integrated cephalostat to avert rotational or angular errors. Special emphasis was placed on cranial symmetry and midline alignment concerning the radiography sensor.

This standardized imaging methodology ensured consistent projection geometry across timepoints, facilitating accurate assessment of skeletal and dental positional alterations. The research attained a high level of inter-timepoint reliability by rectifying anomalies in patient placement and exposure settings, so assuring that the reported cephalometric changes represented genuine anatomical variances rather than technical discrepancies.

Radiographic Image Acquisition

Lateral cephalometric radiographs were acquired by certified radiographic technicians in accordance with standard clinical imaging protocols. Natural head position (NHP) is a consistent and repeatable orientation of the head in an upright posture, with the eyes directed towards a distant point at eye level, indicating that the visual axis is horizontal [35].

All images were obtained using a PaX-i3D Green system (Vatech Co., Ltd., Hwaseong, Republic of Korea; 2019 model), a hybrid digital cephalometric and CBCT unit recognized for its ability to deliver high-resolution imaging at a reduced radiation dose.

Exposure parameters were standardized across all patients to maintain diagnostic consistency: tube voltage 70–90 kVp, tube current 5–10 mA, and exposure time 1.0–1.5 s. The resulting images exhibited an isotropic voxel size ranging from 0.2 to 0.3 mm, depending on patient morphology and selected acquisition mode.

Each cephalogram was exported in JPEG format and subsequently uploaded to the WebCeph cloud-based platform for digital landmark identification, cephalometric tracing, and automated parameter computation.

Although NemoFAB can perform three-dimensional virtual surgical simulations, the current study used standardized lateral cephalometric radiographs for postoperative validation. In the absence of specific clinical indications, routine postoperative CBCT acquisition was not ethically justified in accordance with the ALARA principle and institutional radiation protection protocols. Because cephalometric variables primarily reflecting sagittal and vertical changes have shown high repeatability in both two-dimensional and three-dimensional studies, quantitative analysis was purposefully limited to these variables. The statistical analysis purposefully did not include parameters that were primarily affected by roll, yaw, or transverse discrepancies.

Initial Evaluation and Patient Information

Patients presented either to the orthodontist or the oral and maxillofacial surgeon. Regardless of the first specialist consulted, when a complex interdisciplinary treatment was indicated, each patient was evaluated by both specialists before establishing the treatment plan.

During the first visit, patients completed informed consent forms and general medical questionnaires, followed by an initial consultation. The treatment protocol, including the orthodontic and surgical phases, duration, objectives, and expected outcomes, was explained in detail. Illustrative clinical cases were presented to facilitate understanding of the treatment process.

Data Collection and Diagnostic Work-Up

Comprehensive clinical and radiographic documentation was obtained for each patient, including

- Intraoral and extraoral (cervicofacial) photographs. Standardized extraoral and intraoral photographs were acquired using a full-frame mirrorless digital camera (Nikon Z6 II, Nikon Corporation, Tokyo, Japan) equipped with a 105 mm macro lens (Nikkor Z MC 105 mm f/2.8 VR; Nikon Corporation, Tokyo, Japan). All images were obtained under controlled lighting, using the same photographic protocol to ensure reproducibility.
- Digital impressions of both arches (upper, lower, and in occlusion)
- Specific radiographs depending on the case: panoramic radiograph (OPG), lateral and frontal cephalograms, and CBCT scans (large field of view).

All data were processed and analyzed using WebCeph software for cephalometric interpretation. Skeletal and dental diagnoses were established based on the combined clinical and radiographic findings, followed by elaboration of an individualized treatment plan and estimation of treatment duration.

Treatment Planning and Patient Counseling

A second consultation was scheduled to discuss the diagnosis, proposed treatment plan, and therapeutic alternatives, including associated risks, benefits, and potential complications. After all questions were addressed, patients signed the informed consent to begin active treatment.

Pre-Orthodontic Phase

Prior to appliance placement, all patients underwent professional dental cleaning and resolution of existing odontogenic issues, as required.

Pre-Surgical Orthodontic Treatment

Fixed orthodontic appliances were bonded on both arches (metallic or sapphire brackets according to patient preference). Follow-up appointments were scheduled every 5 weeks for progressive archwire activation. Sequential archwires were used—initially nickel–titanium, followed by stainless steel up to a final rectangular 0.019 × 0.025-inch archwire.

The objective of this phase was dental decompensation, aligning teeth correctly within their skeletal bases to prepare for surgical repositioning of the jaws in all three planes of space (vertical, sagittal, and transverse).

Virtual Surgical Planning

Upon completion of the orthodontic decompensation phase, new records were obtained: updated photographs, intraoral scans, and CBCT (large field of view).

Virtual surgical planning was carried out using NemoFAB (Biotech Dental) software. DICOM and STL files were imported, and 3D models were generated to perform osteotomies virtually. The surgical movements were guided by William Arnett’s soft- and hard-tissue analysis.

Intermediate and final surgical splints were digitally created, exported in STL format, and 3D printed. Their intraoral accuracy was verified preoperatively. Patients then underwent anesthetic evaluation and completed preoperative laboratory testing.

Surgical Procedure

To guarantee unhindered access to the oral cavity, all surgeries were carried out under general nasotracheal anesthesia. The complexity of the case, the number of jaws operated on, the segmentation pattern, and the inclusion or exclusion of genioplasty all affected how long the procedure took. Every patient received standard orthognathic surgical procedures based on their unique dentofacial deformities. A bilateral sagittal split osteotomy (BSSO) was carried out for mandibular correction, and a Le Fort I osteotomy was utilized for maxillary repositioning. Bimaxillary procedures were performed in the same surgical session when necessary. Using titanium plates and screws in accordance with accepted clinical procedures, rigid internal fixation was accomplished in every instance. All procedures were carried out by the same surgical team, which consisted of two skilled oral and maxillofacial surgeons, adhering to a standardized surgical and postoperative protocol in order to reduce variation in surgical technique.

Postoperative Management

On the first postoperative day, two control radiographs were obtained (panoramic and lateral cephalogram), and intermaxillary elastics were applied to guide occlusion. Patients received detailed oral hygiene and dietary instructions, as well as pharmacologic prescriptions (antibiotics, anti-inflammatories, and analgesics). Sutures were removed approximately 14 days post-surgery.

The postoperative orthodontic phase lasted an average of six months, after which retention devices (fixed or removable) were applied. Follow-up appointments were scheduled at 2 months, 6 months, and annually thereafter.

**Figure 1:**
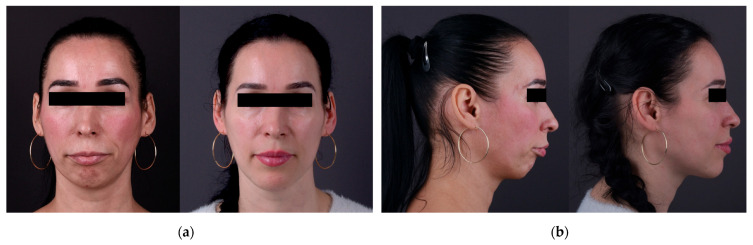

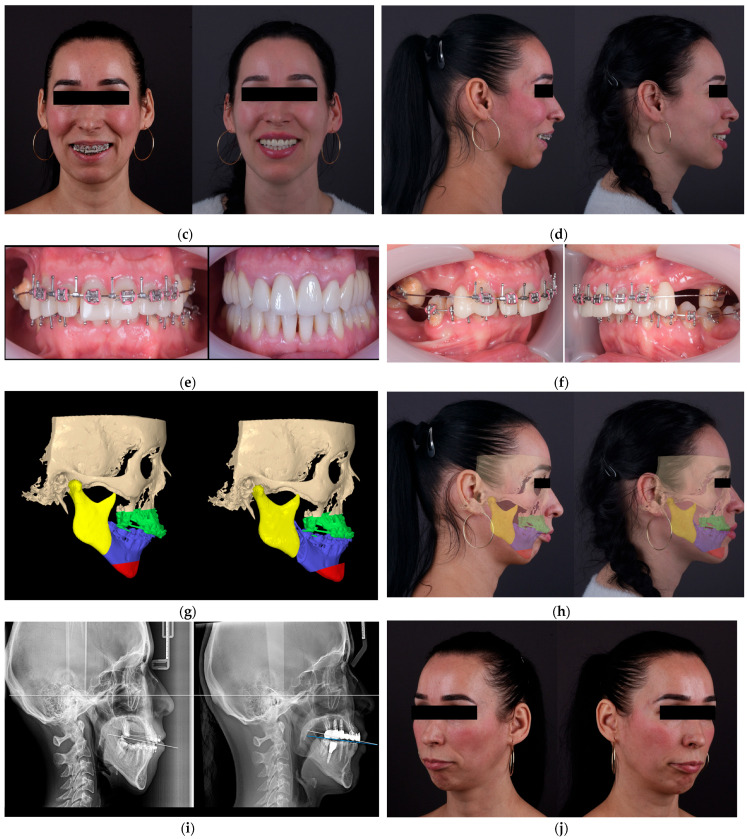
Comprehensive digital planning and clinical results of a skeletal Class II patient undergoing genioplasty and bimaxillary orthognathic surgery under NemoFAB software guidance. (**a**) Frontal facial photos taken prior to orthognathic surgery (**left**) and following it (**right**). The preoperative picture shows mandibular retrognathia and a skeletal Class II pattern. Following mandibular advancement, the maxillo-mandibular complex is rotated counterclockwise, and advancement genioplasty is performed as digitally planned, as seen in the postoperative image. (**b**) Lateral facial photos taken prior to orthognathic surgery (**left**) and following it (**right**). Mandibular retrusion and increased facial convexity are visible in the preoperative profile. The postoperative profile shows skeletal repositioning after advancement genioplasty, counterclockwise rotation of the maxillo-mandibular complex, and digitally planned mandibular advancement. (**c**) Frontal smiling photos taken prior to (**left**) and following orthognathic surgery (**right**). The pictures show how the appearance of the smile changed after bimaxillary orthognathic surgery carried out in accordance with the virtual surgical plan. (**d**) Semi-profile pictures taken prior to (**left**) and following orthognathic surgery (**right**). While the postoperative image shows changes in facial contour after the intended skeletal movements, the preoperative image emphasizes mandibular retrusion and increased facial convexity. (**e**) Intraoral frontal views prior to (**left**) and following (**right**) orthodontic surgery. The preoperative image displays a Class II molar relationship, increased overjet, and dental crowding. The occlusal relationship attained following combined orthodontic and surgical treatment is shown in the postoperative image. (**f**) Intraoral lateral views taken before orthodontic surgery. The pictures show upper incisor proclination, increased overjet, and Class II molar and canine relationships before surgical correction. To get ready for the anticipated skeletal movements, orthodontic decompensation was carried out. (**g**) NemoFAB software for three-dimensional virtual surgical planning. Skeletal segmentation and planned osteotomies for orthognathic correction are depicted in 3D reconstructions derived from CBCT data. The maxilla (yellow), mandibular body and rami (blue), dentoalveolar segments (green), and genioplasty segment (red) are represented by the color-coded components. The mandibular advancement, counterclockwise rotation of the maxillo-mandibular complex, and chin advancement are digitally planned to achieve the intended skeletal repositioning and corresponding soft-tissue changes. (**h**) Integration of hard and soft tissues in virtual surgical planning. To better visualize planned surgical movements, 3D skeletal models are superimposed on facial photos in composite lateral views. Mandibular retrognathia and facial convexity are highlighted in the preoperative image (**left**), whereas advancement genioplasty, counterclockwise rotation of the maxillo-mandibular complex, and mandibular advancement are shown in the postoperative simulation (**right**). This overlay, which is digitally simulated in NemoFAB, shows the relationship between skeletal repositioning and anticipated soft-tissue outcomes. The overlay illustrates correspondence between digitally planned skeletal movements and postoperative findings. (**i**) Lateral cephalometric radiographs taken before and after surgery. The preoperative radiograph (**left**) shows a steep occlusal plane, increased facial convexity, and mandibular retrognathia, all of which are indicative of skeletal Class II discrepancy. Following orthognathic surgery, the postoperative radiograph (**right**) shows improved chin projection, counterclockwise rotation of the maxillo-mandibular complex, and mandibular advancement. The restored maxillo-mandibular alignment and altered occlusal plane attest to the precision of the digitally guided surgical plan carried out using NemoFAB. (**j**) Semi-profile pictures taken prior to (**left**) and following orthognathic surgery (**right**). The preoperative picture demonstrates the skeletal Class II deformity’s mandibular retrusion, increased lower facial convexity, and decreased chin projection. After mandibular advancement, counter-clockwise rotation of the maxillo-mandibular complex, and advancement genioplasty digitally planned using NemoFAB, the postoperative image demonstrates improved lower third proportions, changes in lower facial proportions, and a smoother transition between the lips and chin region.

Figure 1 shows the complete digital planning and clinical documentation of a skeletal Class II patient who underwent genioplasty and bimaxillary orthognathic surgery using NemoFAB virtual surgical planning.

**Figure 2:**
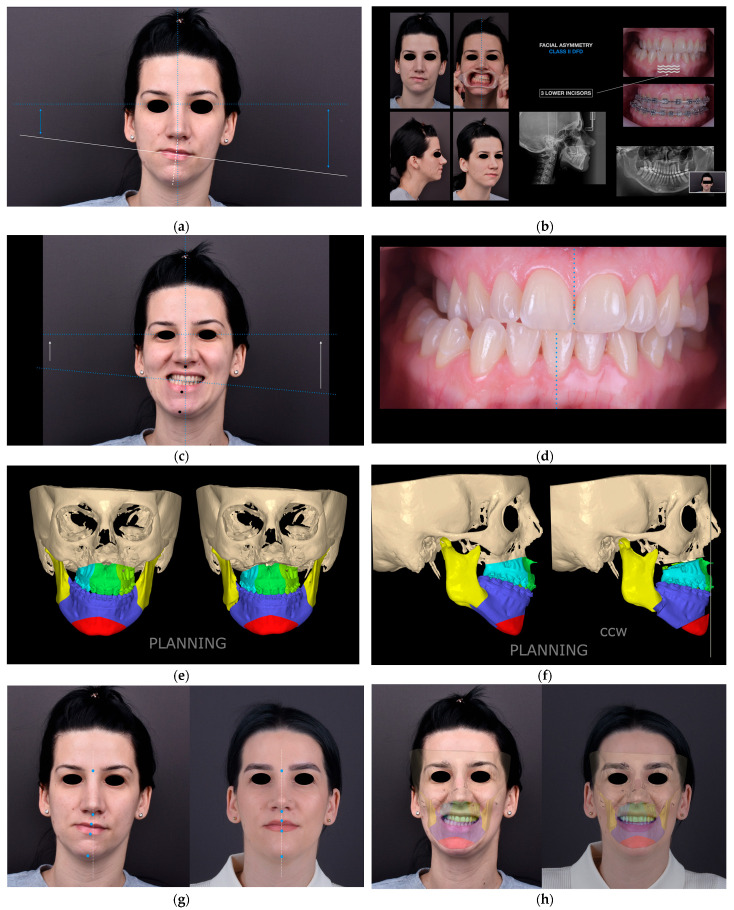

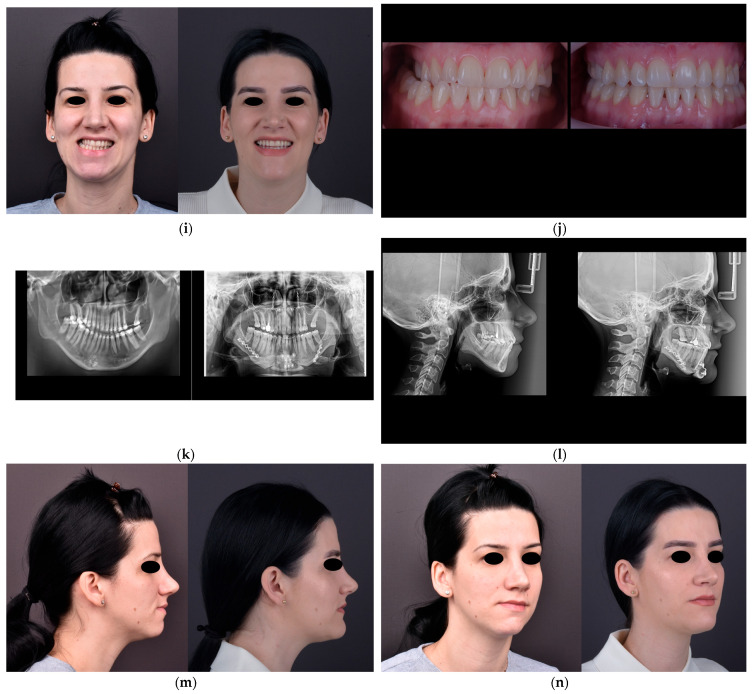
Digital diagnosis, virtual surgical planning, and postoperative documentation of a patient undergoing genioplasty and bimaxillary orthognathic surgery for skeletal Class II dentofacial deformity and facial asymmetry. (**a**) Preoperative frontal facial analysis showing occlusal cant deviation and facial asymmetry. The patient has a right-sided posterior crossbite (inverse occlusion) and a laterodeviated facial midline. An occlusal cant and vertical asymmetry between the left and right sides of the face are revealed by the analysis. There is a discernible asymmetry and imbalance in the lower face due to the mandibular deviation to the right. (**b**) Detailed preoperative diagnostic records of the patient with skeletal Class a II dentofacial deformity (DFD) and facial asymmetry. A laterodeviated mandible, Class II skeletal relationship, and facial asymmetry are all visible in clinical photos and radiographs. Three lower incisors and a right-sided posterior crossbite are visible in intraoral views, which add to the skeletal and dental imbalance. The patient’s Class II DFD diagnosis is supported by cephalometric and panoramic radiographs, which also show an asymmetric occlusal plane and mandibular retrusion. (**c**) Analysis of frontal smiling reveals occlusal cant and facial asymmetry. The mandible and chin point are moved to the right in the preoperative frontal smiling photo, which shows a deviation of the dental and facial midlines. An underlying skeletal asymmetry is highlighted by the occlusal cant and uneven gingival display between the two sides. In order to correct the midline deviation and restore facial balance, these parameters were crucial points of reference during digital planning. (**d**) Intraoral frontal view demonstrating lower incisor asymmetry and dental midline deviation. The preoperative intraoral image reveals anterior crowding that contributes to the arch asymmetry, a mandibular dental midline deviation to the right, and only three lower incisors. In order to achieve appropriate occlusal and facial alignment, the difference between the maxillary and mandibular midlines—which reflects the underlying skeletal asymmetry—was carefully taken into account during digital orthodontic and surgical planning. (**e**) Digital orthognathic surgical planning from the front. The virtual simulation shows how the maxillo-mandibular complex can be repositioned to create an occlusal plane that is parallel to the ground and horizontally aligned. Through multisegmentation of the maxilla (three-piece Le Fort I osteotomy), the right-sided posterior crossbite was treated, enabling transversal expansion and the restoration of appropriate transverse occlusion and symmetry. (**f**) Sagittal view of the digital orthognathic surgical planning showing the maxillo-mandibular complex rotating counterclockwise. The virtual plan shows a counterclockwise rotation of the maxillo-mandibular complex (CCW) in the sagittal (antero-posterior) plane to enhance the chin projection, vertical dimension, and facial convexity. In accordance with the digital plan made in NemoFAB, this movement was synchronized with mandibular advancement and genioplasty to achieve ideal skeletal harmony and soft-tissue balance. (**g**) Frontal photos taken before (**left**) and after (**right**) orthognathic surgery, demonstrating the correction of midline deviation and facial asymmetry. Mandibular laterodeviation is consistent with the preoperative image’s chin asymmetry and facial midline deviation to the right. The accuracy of the digitally guided surgical plan is confirmed by the postoperative photo, which displays a centered facial and dental midline, symmetrical lower facial thirds, and postoperative facial changes following digitally guided surgery. (**h**) Preoperative and postoperative facial photos superimposed taken before (**left**) and after (**right**) orthognathic surgery to show the relationship between clinical outcome and digital planning. The overlays show that the skeletal and soft-tissue movements that were digitally planned were achieved postoperatively after surgery. The successful translation of the virtual surgical plan into the final clinical outcome is confirmed by the alignment of facial midlines, correction of the occlusal plane, and restoration of symmetry. (**i**) Frontal smiling photos taken before (**left**) and after (**right**) orthognathic surgery, demonstrating changes in smile characteristics and facial symmetry. Due to skeletal and dental imbalance, the preoperative image shows uneven gingival display and asymmetrical smile dynamics. The postoperative photo shows a symmetrical, harmonious smile with balanced gingival exposure and improved facial expression, demonstrating the orthognathic treatment’s functional and aesthetic success. (**j**) Intraoral frontal views demonstrating occlusal correction and dental alignment taken before (**left**) and after (**right**) orthodontic–surgical treatment. With just three lower incisors, the preoperative image shows anterior crowding, dental midline deviation, and Class II malocclusion. After orthognathic surgery and orthodontic finishing, the postoperative photo shows perfect intercuspation, dental midline coincidence, and harmonious arch coordination, indicating postoperative occlusal and dental alignment. (**k**) Panoramic radiographs showing skeletal correction and fixation taken before (**left**) and after (**right**) surgery. Three lower incisors, a Class II skeletal relationship, and mandibular asymmetry are all visible on the preoperative radiograph. The successful execution of the digitally planned surgical movements and precise maxillo-mandibular repositioning is confirmed by the postoperative panoramic image, which shows stable osteosynthesis with fixation plates and screws at the Le Fort I, bilateral sagittal split osteotomy (BSSO), and genioplasty sites. (**l**) Lateral cephalometric radiographs showing sagittal skeletal correction taken before (**left**) and after (**right**) surgery. The preoperative radiograph shows a steep occlusal plane, increased facial convexity, and a skeletal Class II relationship with mandibular retrusion. In line with the digital surgical plan, the postoperative image shows mandibular advancement, counterclockwise rotation of the maxillo-mandibular complex, and forward repositioning of the chin, producing a balanced skeletal profile and harmonized facial proportions. (**m**) Lateral facial photos demonstrating improved profiles taken before (**left**) and after (**right**) orthognathic surgery. The preoperative image shows a deep labiomental fold, increased facial convexity, and mandibular retrusion, all of which are indicative of a skeletal Class II profile. Postoperative facial profile changes consistent with the digital surgical plan are observed in the postoperative photo, which also shows improved chin projection, a reduction in the depth of the labiomental fold, and a harmonized facial contour. (**n**) Semi-profile photos showing improved facial harmony taken before (**left**) and after (**right**) orthognathic surgery. Mandibular retrusion, flattened midface projection, and increased facial convexity—all indicative of skeletal Class II deformity—are visible on the preoperative image. The postoperative image illustrates postoperative facial changes in the digitally planned surgical correction by showing improved lower facial projection, a smoother facial contour, and a more proportionate relationship between the upper, middle, and lower facial thirds.

A representative clinical case illustrating the complete digital diagnostic workflow, virtual surgical planning, and postoperative outcomes is presented in Figure 2. The figure demonstrates the correction of facial asymmetry and occlusal cant through digitally guided bimaxillary orthognathic surgery, highlighting the correspondence between preoperative diagnosis, virtual planning, and postoperative clinical results.

**Figure 3:**
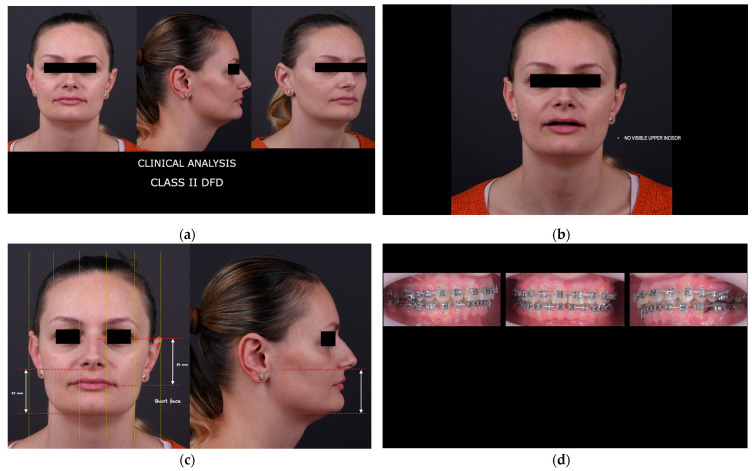

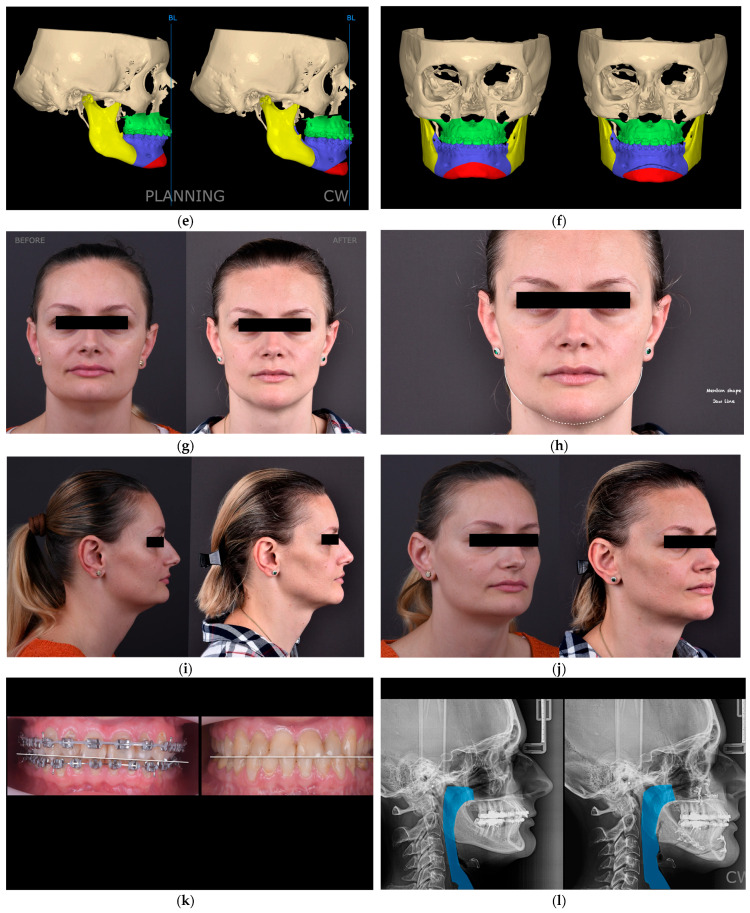

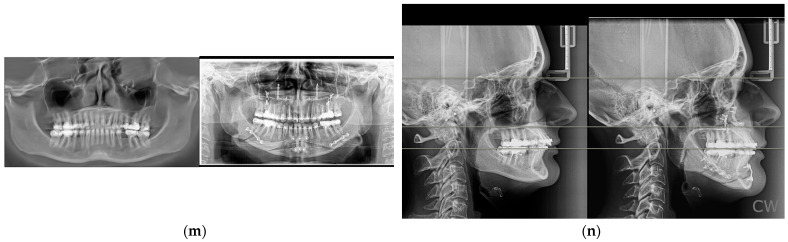
Digital planning and postoperative clinical documentation for orthognathic correction of a skeletal Class II short-face pattern using genioplasty and clockwise rotation of the maxillo-mandibular complex. (**a**) Preoperative clinical analysis of a patient with a skeletal Class II dentofacial deformity, displaying frontal, lateral, and oblique facial views. In line with a Class II skeletal pattern, the pictures show mandibular retrusion, increased facial convexity, and decreased lower facial projection. (**b**) A patient with a skeletal Class II short-face pattern has a reduced upper incisor display in this preoperative frontal facial photograph taken at rest. The picture shows a shorter lower face and less exposure of the maxillary incisors when at rest. (**c**) A vertical deficiency consistent with a short-face morphology is shown by frontal and lateral facial proportional analysis. It is clear that the lower facial height has decreased, and digital surgical planning took this into account. (**d**) Intraoral photos taken prior to orthodontic decompensation surgery. Prior to surgical correction, frontal and lateral views reveal fixed orthodontic appliances, Class II molar and canine relationships, increased overjet, and retroclined lower incisors. (**e**) A sagittal view of the NemoFAB software-based digital orthognathic surgical planning. Skeletal segmentation, planned clockwise rotation of the maxillo-mandibular complex, mandibular repositioning, and genioplasty are all depicted in three-dimensional models. The maxilla (green), mandibular body and rami (yellow and blue), and genioplasty segment (red) are represented by color-coded segments. (**f**) Digital orthognathic surgical planning from the front. The intended vertical repositioning and clockwise rotation of the maxillo-mandibular complex after orthodontic decompensation are shown in the virtual simulation. (**g**) Frontal facial photos taken prior to orthognathic surgery (**left**) and following it (**right**). Following the maxillo-mandibular complex’s clockwise rotation and vertical repositioning in accordance with the digital plan, the images capture changes in the face. (**h**) A postoperative frontal facial photo taken after surgery (**right**) showing the mandibular contour changes after genioplasty and orthognathic surgery. (**i**) Lateral facial photos taken prior to (**left**) and following orthognathic surgery (**right**). Following mandibular repositioning and clockwise rotation of the maxillo-mandibular complex, the postoperative image records changes in the profile. (**j**) Semi-profile photos of the face taken prior to (**left**) and following orthognathic surgery (**right**). After digitally planned skeletal repositioning, the pictures show changes in facial contour. (**k**) Intraoral frontal views taken before (**left**) and after (**right**) orthognathic surgery. While the postoperative image shows the occlusal relationship attained after skeletal repositioning, the preoperative image shows occlusal canting. (**l**) Lateral cephalometric radiographs taken before surgery (**left**) and after surgery (**right**). The pictures show the maxillo-mandibular complex rotating clockwise after orthognathic surgery and mandibular advancement. The current study did not quantitatively evaluate changes in airway dimensions. (**m**) Panoramic radiographs taken before surgery (**left**) and after surgery (**right**). Following digitally guided surgery, the postoperative image displays fixation with plates and screws at the Le Fort I osteotomy, bilateral sagittal split osteotomy (BSSO), and genioplasty sites. (**n**) Preoperative and postoperative lateral cephalometric radiographs taken before (**left**) and after (**right**) surgery showing changes in the orientation of the occlusal plane after clockwise rotation of the maxillo-mandibular complex.

A representative clinical case of a skeletal Class II short-face pattern treated with digitally guided orthognathic surgery is illustrated in Figure 3. The figure highlights the integration of preoperative clinical assessment, virtual surgical planning involving clockwise rotation of the maxillo-mandibular complex, and postoperative clinical and radiographic outcomes, demonstrating the correspondence between the digital plan and the achieved surgical result.

Preoperative evaluation, virtual surgical planning, and postoperative clinical documentation of a patient undergoing digitally guided orthognathic surgery for a skeletal Class II dentofacial deformity and short face pattern.

### 2.2. Statistical Analysis

All statistical analyses were conducted in R (version 4.3.1) using RStudio (version 2023.09.1; RStudio PBC, Boston, MA, USA). Continuous variables were initially checked for data-entry errors and outliers, then summarized as mean and standard deviation (mean (SD)) when approximately normally distributed, and as median and interquartile range (median (Q1, Q3)) when distributional assumptions were not met. Categorical variables were described as n (%). Normality of continuous data was assessed with Shapiro–Wilk tests and visual inspection of histograms and Q–Q plots. For each continuous variable, a one-way repeated-measures analysis of variance (RM-ANOVA) was applied to compare the three measurement conditions (preoperative, Nemo, and postoperative), with Greenhouse–Geisser corrections to the degrees of freedom when the sphericity assumption was violated, and results are presented with F-statistics and corresponding *p*-values. For variables with a statistically significant global effect, planned pairwise post hoc comparisons were performed using paired *t*-tests (Pre vs. Post, Pre vs. Nemo, Nemo vs. Post), with Bonferroni-adjusted *p*-values to correct for multiple testing. Effect sizes for pairwise comparisons were quantified using Cohen’s d for dependent samples. For a more detailed analysis of change over time, paired *t*-tests comparing preoperative and postoperative measurements and preoperative and Nemo measurements were performed, with mean differences, t-statistics, two-sided *p*-values, and Cohen’s d for each comparison calculated, along with descriptive means and SDs for both time points. The agreement between clinical measurements and Nemo predictions, and between time points, was evaluated by Bland–Altman analysis and intraclass correlation coefficients (ICC).

## 3. Results

The study sample consisted of adults with a median age of 29.0 years (IQR: 26.0–34.0), indicating a relatively young cohort typical for orthognathic and orthodontic treatment. The distribution of sex was balanced, with 19 (48%) females and 21 (53%) males.

Analysis of the dental variables revealed significant differences between the three time points, especially in parameters related to sagittal and vertical occlusive patterns. Overdose showed significant reductions from pre-operative to post-operative measurements, and the Nemo prognostic model was close to the final post-operative condition as reflected in the highly significant ANOVA (F = 13.66, *p* < 0.00001). Overbite followed a similar pattern and showed a significant overall difference (F = 6.942, *p* = 0.00168), although the postoperative values showed a more pronounced reduction than the mid-range estimate of the Nemo score. In the case of dental axial tilt, the position of the maxillary incisor relative to the occlusive plane showed a significant difference between conditions (F = 16.474, *p* < 0.000002). Both the Nemo and postoperative values were above preoperative ranges, indicating that surgical repair and virtual planning predicted similar post-treatment orientations. The inclination of the mandibular incisors did not change significantly between conditions (F = 0.699, *p* = 0.500), suggesting stable mandibular anterior dento-alveolar positioning regardless of surgical intervention or predictive modelling. Exposure to the maxillary incisor showed significant variability (F = 12.066, *p* < 0.00003), and the postoperative and Nemo values reflected decreased gingival imaging compared to pre-op measurements. On the other hand, maxillary height remained statistically stable (F = 0.205, *p* = 0.815), indicating that both surgical results and virtual predictions maintained the vertical positioning of the maxilla throughout the entire study. Several skeletal projections also showed significant differences. The position of the maxillary incisors relative to the true vertical line (TVL) varied significantly between conditions (F = 5.701, *p* = 0.004886), with the Nemo procedure predicting a posterior projection more anteriorly than pre- and post-operative. A more pronounced trend was seen in mandibular incisors (F = 31.419, *p* < 0.000000001), where both postoperative and baseline Nemo values showed significant posterior displacement, which is closely correlated. For skeletal markers, the B-point projection was close to statistical significance (F = 2.527, *p* = 0.086), indicating relative stability over time. The prognostic projections showed no significant change (F = 0.147, *p* = 0.863), suggesting that the chin position remained consistent pre- and post-operative. Finally, the angle of the occlusive plane to the TVL showed significant variation (F = 16.08, *p* < 0.000002), with a downward shift in both the Nemo and postoperative inclined planes in relation to preoperative orientations (Table 1).

Comparison of preoperative and postoperative measurements revealed significant modifications in several dentoalveolar and skeletal parameters following treatment. Overjet demonstrated a substantial reduction, decreasing from a preoperative mean of 5.28 (SD 2.65) to 3.30 (SD 1.59), with a mean difference of 1.981 and a statistically significant t-value of 3.816 (*p* = 0.000472). The associated Cohen’s d of 0.603 indicates a moderate-to-large effect, suggesting clinically meaningful correction. Overbite followed a similar pattern, declining from 3.06 (SD 2.17) to 1.86 (SD 1.29), corresponding to a mean reduction of 1.202 (t = 3.149, *p* = 0.003138) and a moderate effect size (d = 0.498), reflecting a consistent improvement in vertical dental overlap.

For dental axial inclination, the maxillary incisor position relative to the maxillary occlusal plane increased significantly postoperatively, shifting from 57.15 (SD 4.16) to 60.71 (SD 5.75). The mean difference of −3.56, supported by a t-statistic of −4.543 and *p* < 0.0001, alongside a Cohen’s d of −0.718, indicates a large and clinically relevant change. Conversely, the mandibular incisor inclination exhibited no significant difference between preoperative and postoperative values, with a mean change of only 0.883 (t = 0.637, *p* = 0.528), reflecting a negligible effect size (d = 0.101) and indicating relative stability of mandibular incisor angulation across treatment.

Maxillary incisor exposure decreased from 3.93 (SD 2.58) preoperatively to 2.34 (SD 2.14) postoperatively, yielding a significant mean difference of 1.586 (t = 3.955, *p* = 0.000313) and a moderate-to-large effect size (d = 0.625). In contrast, maxillary height demonstrated no meaningful change across treatment, with a modest and nonsignificant mean difference of −0.621 (t = −1.229, *p* = 0.227), accompanied by a small effect size (d = −0.194).

Skeletal projection parameters revealed striking differences in some areas while remaining stable in others. The maxillary incisor projection to the TVL showed a trend toward improvement, with a mean difference of −1.307; however, this change approached but did not reach statistical significance (t = −1.911, *p* = 0.06344), and the effect size remained small (d = −0.302). In contrast, a highly significant posterior repositioning of the mandibular incisor relative to the TVL was observed, shifting from −8.16 (SD 8.28) to −15.84 (SD 4.21). The mean difference of 7.673, combined with a t-statistic of 5.932 (*p* < 0.0000007) and a large effect size (d = 0.938), underscores a robust and clinically important change that aligns with expected surgical correction.

However, skeletal landmarks such as B-point and Pogonion demonstrated minimal and statistically nonsignificant differences. B-point changed by only 0.761 (t = 0.886, *p* = 0.381; d = 0.140), and Pogonion by 0.217 (t = 0.222, *p* = 0.826; d = 0.035), indicating a high degree of postoperative positional stability for these anatomical structures.

Finally, the maxillary occlusal plane angle to the TVL decreased significantly from 98.71 (SD 5.29) to 95.92 (SD 4.29), with a mean difference of 2.788 (t = 3.442, *p* = 0.00139) and a moderate effect size (d = 0.544). This reflects a consistent rotational adjustment of the maxillary occlusal plane following surgical intervention (Table 2).

A comparison of preoperative clinical measurements and Nemo-derived virtual predictions revealed different patterns of agreement and differences in the dental and skeletal parameters assessed. These differences reflect both the predictive capability of the Nemo system and the inherent anatomical variability that preoperative conditions present. Dental alveolar parameters showed a variable correspondence between preoperative and postoperative measurements. The excess weight decreased from 5.28 (SD 2.65) pre-op to 3.50 (SD 1.00) in the Nemo analysis, with a significant median difference of 1.776 (t = 3.692, *p* = 0.000680) and a moderate magnitude of effect (d = 0.584), suggesting that Nemo consistently predicts a reduction in the anteroposterior dental deficit. The magnitude of the effect was smaller, from 3.06 (SD 2.17) to 2.44 (SD 1.15), with an average difference of 0.621, but it was not statistically significant (t = 1.613, *p* = 0.114796), and the magnitude of the effect was small (d = 0.255). These findings indicate that vertical dento-alveolar changes are less accurately predicted by virtual simulation than sagittal changes. In the Nemo analysis, the angle of dental axial inclination relative to the occlusal plane increased significantly, from 57.15 (SD 4.16) to 61.11 (SD 7.41). The mean difference of -3.951 was statistically significant (t = −4.082, *p* = 0.000214), and the magnitude of the effect was moderate to large (d = −0.645), suggesting that Nemo tends to model a more prominent position of the maxillae incisors than preoperatively. In contrast, mandibular incisor angulation was stable in both conditions, with only a minor magnitude of effect (t = 0.919, *p* = 0.145) between the preoperative mean of 72.84 (SD 11.92) and the Nemo value of 72.45 (SD 7.87). This stability indicates that the mandibular anterior axial tilt has not been significantly altered by Nemo’s simulations. Soft tissue and skeletal parameters showed more pronounced differences. Exposure to maxillary incisors decreased slightly from 3.93 (SD 2.58) to 2.93 (SD 1.36), with a significant median difference of 1000 (t = 2.795, *p* = 0.008016) and moderate magnitude of effect (d = 0.442). However, the maxillary height remained very consistent between conditions, with a negligible mean difference of −0.293 (t = −0.246, *p* = 0.807233), which suggests that the vertical position of the maxilla is largely maintained in the Nemo simulation. The projected skeletal features showed larger variations. The median incisor projection relative to the true vertical line varied significantly, from −13.84 (SD 2.81) to −10.96 (SD 6.10), with a significant median difference of −2.886 (t = −3.062, *p* = 0.484). The most significant difference was observed in mandibular incisors, which shifted from −8.16 to −8.28 preoperatively (Table 3).

The Bland–Altman analysis revealed distinct levels of agreement between preoperative measurements, Nemo predictions, and postoperative outcomes. Comparisons between Pre vs. Nemo showed the widest limits of agreement across most parameters, indicating substantial variability and highlighting the anatomical disparities between baseline conditions and virtual predictions. Larger biases were observed in variables such as the Mx1 to Mx plane and Md1 to TVL, suggesting that Nemo anticipates more pronounced skeletal and dentoalveolar corrections than those present preoperatively. The pre- vs. post-comparison demonstrated narrower limits of agreement for several measurements, particularly in overjet, overbite, and maxillary incisor exposure, reflecting the expected clinical improvements following treatment. Nevertheless, certain skeletal projections such as Md1 to TVL continued to exhibit wide limits, indicating the complexity and variability of postoperative skeletal changes. The highest agreement was consistently observed in the Nemo vs. Post comparison. Bias values were small, and limits of agreement were markedly narrower than in other comparisons, especially for overjet, overbite, and most skeletal landmarks. These findings indicate that Nemo predictions closely approximate actual postoperative outcomes for both dentoalveolar and skeletal parameters. The results collectively suggest that Nemo functions as an accurate simulation tool for forecasting postoperative changes, particularly in variables directly influenced by surgical planning (Table 4).

**Figure 4:**
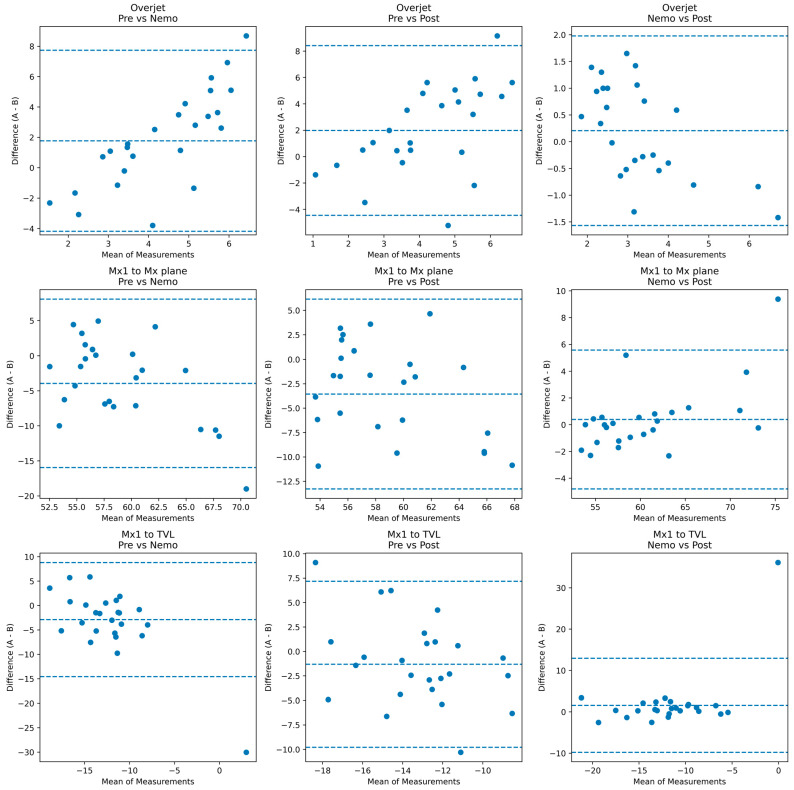
Bland–Altman Plots Comparing Preoperative, Nemo, and Postoperative Measurements Across Key Dental and Skeletal Variables.

The Bland–Altman plots illustrate the degree of agreement between preoperative measurements, Nemo virtual predictions, and postoperative outcomes for selected parameters. Across all variables, the widest dispersion and broadest limits of agreement were observed in the Pre vs. Nemo comparisons, indicating substantial baseline discrepancies between the clinical preoperative state and the anticipated postoperative configuration modeled by the software. The Pre vs. Post comparisons exhibited reduced variability, reflecting the expected clinical correction following surgical intervention. The Nemo vs. Post comparisons consistently demonstrated the smallest biases and the narrowest limits of agreement, indicating that Nemo predictions closely approximate the actual postoperative results, particularly for Mx1 to TVL and Mx1 to Mx plane measurements. Together, these plots confirm that Nemo reliably forecasts the postoperative anatomical configuration, while larger preoperative discrepancies highlight the magnitude of intended surgical correction (Figure 4).

The evaluation of agreement across the three measurement conditions preoperative clinical records, Nemo virtual simulation, and postoperative outcomes reveals a heterogeneous pattern of reliability, with clear distinctions between dentoalveolar measurements, skeletal projections, and occlusal plane assessments.

In the dentoalveolar domain, the global ICC values demonstrate variable but generally acceptable agreement among the three methods. Overjet and Overbite show global ICC values of 0.025 and 0.381, respectively, both falling within the poor range, indicating that preoperative, Nemo, and postoperative measurements do not align closely for these parameters. Pairwise ICC values support this finding, as both Pre–Nemo and Pre–Post comparisons remain poor, with only the Nemo–Post comparison achieving a good level of agreement (ICC = 0.763 for Overjet and ICC = 0.681 for Overbite). These results indicate that Nemo offers a closer approximation to the final postoperative condition than to the baseline preoperative state, reinforcing its relevance as a predictive instrument rather than a diagnostic replica of initial anatomical conditions.

For dental axial inclination measurements, the Mx1 to Mx plane and Md1 to Md plane variables exhibit markedly higher levels of agreement. The global ICC for Mx1 to Mx plane reached 0.816, categorized as good, while Md1 to Md plane achieved 0.864, also within the good range. Notably, the Nemo–Post comparison reached excellent agreement for Mx1 to Mx plane (ICC = 0.921) and Md1 to Md plane (ICC = 0.984), confirming that the postoperative orientation of incisors is reliably anticipated by the Nemo simulation. Pre–Post comparisons yielded moderate agreement, whereas Pre–Nemo agreement remained lower, supporting the interpretation that surgical correction drives the transition from preoperative variability to postoperative consistency, a pattern well captured by Nemo’s predictive modeling.

Soft-tissue and skeletal projection measurements demonstrate a more mixed profile. Maxillary height presented moderate global agreement (ICC = 0.694), although pairwise ICC values indicated poor agreement between Pre–Nemo and poor agreement between Nemo–Post. The Mx1 to TVL and Md1 to TVL projections showed global ICC values of 0.483 and 0.439, respectively, both classified as poor. Pairwise comparisons confirm this limited concordance, with all Pre–Nemo and Pre–Post ICC values in the poor range. Nevertheless, the Nemo–Post comparison for Md1 to TVL achieved excellent agreement (ICC = 0.862), indicating that postoperative mandibular incisor projection is accurately predicted by Nemo despite the low reproducibility seen across preoperative measurements. B-point and Pogonion projections exhibit comparatively stronger reliability. Their global ICC values of 0.830 and 0.857 reflect good agreement among all three measurement conditions, with Nemo–Post analyses reaching good and excellent levels, respectively, thereby suggesting that sagittal skeletal landmarks—particularly at the chin—are modeled with high fidelity by the virtual simulation.

Occlusal plane inclination, represented by Mx plane to TVL, yielded a global ICC of 0.686, indicating moderate agreement. Pairwise ICC values remained poor for most comparisons except for Nemo–Post, which showed moderate reliability (ICC = 0.672). These findings suggest that variations in occlusal plane inclination are less consistently captured across all three measurement environments, but the postoperative condition remains more aligned with the virtual plan than with the initial preoperative state.

Taken together, the ICC results demonstrate that the highest levels of agreement consistently occur between Nemo and postoperative measurements, often reaching good or excellent reliability, particularly for skeletal and dental axial parameters. In contrast, agreement between preoperative and Nemo measurements is uniformly poor, reflecting the expected discrepancy between baseline anatomy and predicted surgical correction. Global ICC values further confirm that variables related to skeletal projection and dental axial inclination show stronger reproducibility across all three modalities, whereas dentoalveolar overjet and overbite values remain more variable. Overall, the pattern indicates that Nemo serves as a reliable predictive tool for postoperative outcomes, particularly for parameters involving skeletal projection and incisor angulation, while preoperative variability limits agreement in parameters influenced by malocclusion severity (Table 5).

## 4. Discussion

Based on the concepts presented in Facial Keys to Orthodontic Diagnosis and Treatment Planning, the Soft Tissue Cephalometric Analysis (STCA) is an organized cephalometric framework. By standardizing reference planes and landmark interpretation and integrating occlusal relationships and facial proportional assessment, it offers an objective technique for soft-tissue evaluation [29].

Treatment planning in orthodontics and orthognathic surgery has been greatly aided by this framework. Over the past 20 years, orthodontics and orthognathic surgery have seen a significant increase in the use of computerized methods for diagnosis and treatment planning. The increasing role of digital workflows in enhancing planning accuracy and reproducibility is highlighted in a sizable body of research published between 2007 and 2017 [7]. According to earlier research, computer-assisted planning makes it easier to correct complex maxillofacial deformities like mandibular repositioning, segment alignment, and yaw discrepancies [6]. According to a number of authors, virtual orthognathic planning helps ensure that the surgical plan is consistently translated to the operating field and permits precise execution of planned skeletal movements [36,37]. The current study concentrated on the quantitative agreement between digitally planned and postoperative dentoskeletal parameters, although previous studies have described patient satisfaction and aesthetic perception. The current study’s results align with earlier institutional research highlighting the benefits of digital integration in dental and surgical workflows [38].

When assessing predictive accuracy across various clinical stages, digital planning systems minimize operator-dependent variability and support standardized measurement protocols. Similar findings about how digital standardization improves reproducibility in the medical and dental fields have been documented [39,40,41]. Predicting clinically significant skeletal and dentoalveolar changes through virtual modeling is a common goal among dental specialties, as evidenced by the increasing use of digital simulation technologies [42]. The current study used repeated measurements and consensus-based landmark identification to lower examiner-related variability. Similar structured evaluation techniques in medical imaging have demonstrated that standardized criteria improve interobserver reliability, supporting the usefulness of predefined measurement frameworks [43].

Adjunctive plaque-control measures may support periodontal stability throughout complex interdisciplinary therapies, and maintaining proper oral hygiene during orthodontic-surgical treatment and postoperative recovery is still crucial [44].

These results are in line with recent research showing that in orthodontics and orthognathic surgery, the combination of advanced three-dimensional facial scanning, combined hard- and soft-tissue analyses, and AI-driven, face-oriented digital workflows enhances diagnostic standardization, reproducibility, and treatment planning accuracy [45,46,47].

AI-assisted cephalometric environments such as NemoFAB may further enhance consistency in this context by enabling consistent landmark placement and measurement procedures. The study’s results demonstrated the highest level of agreement between NemoFAB predictions and postoperative measurements for parameters primarily impacted by sagittal and vertical skeletal repositioning. These findings are in line with previous research showing that three-dimensional simulation systems can replicate postoperative skeletal changes with clinically acceptable accuracy when appropriate reference systems are used [48].

Natural head position and true vertical reference lines are still necessary for accurate interpretation of sagittal and vertical discrepancies, according to Gateno, Xia, and Teichgraeber [49]. The agreement between preoperative and postoperative values varied more for parameters affected by soft-tissue behavior and dentoalveolar adaptation. This variability, which has been frequently reported in the literature [50], may be due to individual differences in soft-tissue thickness, elasticity, muscular tone, and postoperative healing responses. These biological factors are still challenging to accurately simulate and demonstrate the limitations of current predictive algorithms.

Despite these drawbacks, a number of skeletal landmarks showed consistent correspondence between planned and postoperative values, such as mandibular projection and occlusal plane orientation. When paired with structured cephalometric frameworks like STCA, this demonstrates the accuracy of digital planning systems in predicting hard-tissue outcomes. Virtual planning should be understood as a target configuration that directs surgical execution rather than a direct replication of baseline anatomy.

Clinical Implications

The results of this study have important ramifications for orthognathic surgery and clinical orthodontics. The regular use of virtual planning in complex dentofacial cases is supported by the observed agreement between NemoFAB predictions and postoperative measurements for specific dentoskeletal parameters, especially for movements involving sagittal and vertical repositioning. These findings are in line with earlier research showing how three-dimensional planning tools can increase surgical accuracy and predictability [48,49,50]. A comprehensive method that combines objective cephalometric measurements with customized treatment planning is provided by integrating STCA-based assessment with virtual surgical planning. However, the observed variability in soft-tissue dynamics-influenced parameters highlights the need for cautious interpretation of expected results in clinical settings where postoperative adaptation may differ. By offering a consistent visual depiction of intended skeletal modifications, accurate digital simulations may also facilitate interdisciplinary communication and patient counseling. Clinicians should make clear the limitations related to biological variability and soft-tissue prediction, even though these tools can improve understanding of treatment objectives. With documented effects on function and quality of life, dento-maxillary anomalies are a major public health concern, according to epidemiological data [51,52]. AI-assisted cephalometric system integration may enhance planning consistency and diagnostic accuracy, supporting more successful treatment approaches for dento-maxillofacial abnormalities [53]. While highlighting the significance of parameter-specific interpretation and recognition of biological limitations, these findings collectively support the ongoing integration of AI-driven cephalometrics and virtual planning systems into orthognathic workflows.

Study Limitations and Future Perspectives

Although there are a number of limitations to take into account when interpreting the results, this study offers insightful information about the predictive performance of NemoFAB in orthognathic surgery planning.

First, the single-center design and relatively small cohort may limit the generalizability of the results to populations with different craniofacial features or ethnic backgrounds, even though the sample size was sufficient for the applied statistical analyses. To increase external validity and create population-specific reference standards, future multicenter studies with bigger and more varied sample sizes are needed. Second, while NemoFAB creates three-dimensional virtual surgical simulations, postoperative validation was carried out using two-dimensional lateral cephalometric radiographs. Although 2D cephalometry is still a common and clinically useful follow-up technique, it restricts the evaluation of transverse and rotational accuracy. As a result, sagittal and vertical dentoskeletal parameters are the main subjects of the reported predictive validity. To achieve thorough spatial validation, future research utilizing surface-based three-dimensional superimposition, volumetric analyses, and postoperative cone-beam computed tomography (CBCT) is necessary.

Third, despite the use of skilled examiners and consensus procedures, cephalometric landmark identification required a manual verification process that could introduce operator-dependent variability. Future research may see a decrease in subjectivity and an improvement in measurement consistency if artificial intelligence-driven automated or semi-automated landmark detection systems are developed further.

Furthermore, because of individual variations in tissue elasticity, muscular adaptation, and postoperative healing, the soft-tissue response to skeletal repositioning is still intrinsically variable. Predictive accuracy may be further improved by refining soft-tissue biomechanical models and incorporating patient-specific tissue characteristics. Lastly, the current study did not assess long-term stability; instead, it concentrated on short- to mid-term postoperative outcomes. The results of orthognathic treatment may change over time as a result of soft-tissue adaptation and skeletal remodeling. To determine whether digitally planned movements stay stable and whether virtual prediction models can accurately predict long-term changes, longitudinal studies are required. Furthermore, future thorough analyses should take into account other clinically significant parameters that were not assessed, such as condylar position, airway dimensions, transverse skeletal changes, and temporomandibular joint adaptation. Notwithstanding these drawbacks, it is anticipated that further advancements in artificial intelligence, sophisticated soft-tissue modeling, and sizable population-based datasets will improve the precision, repeatability, and clinical usefulness of virtual surgical planning systems in orthognathic surgery.

Certain spatial relationships may be distorted by two-dimensional projections, and their capacity to capture transverse and volumetric discrepancies—which are important in orthognathic surgery—is limited. As a result, 2D cephalometric imaging is less reliable for evaluating parameters that are mainly affected by facial asymmetry or yaw and roll rotations.

The clinical heterogeneity of the sample, which included patients with a range of dentofacial abnormalities, is another drawback of the current investigation. Despite reflecting actual orthognathic practice, the statistical power was insufficient to allow for meaningful subgroup analyses based on particular skeletal patterns. Therefore, rather than being interpreted as deformity-specific accuracy, the results should be interpreted as an overall evaluation of NemoFAB’s predictive performance across a heterogeneous orthognathic population. To examine predictive accuracy based on particular skeletal classifications and surgical movement patterns, future research with larger, stratified cohorts is necessary.

The type of dentofacial deformity and the complexity of the surgical movement can affect prediction accuracy. Specifically, asymmetric cases and procedures involving multi-segment maxillary osteotomies or yaw or roll correction may introduce more variability and be less accurately captured by two-dimensional postoperative validation. The current results should be interpreted as an overall evaluation of NemoFAB’s predictive performance across a heterogeneous orthognathic population because the sample size within particular deformity subgroups was insufficient for sufficiently powered analyses. Future research examining deformity-specific predictive accuracy with larger, stratified cohorts is necessary.

Variability in postoperative outcomes may have been caused by subtle variations in surgical execution and individual postoperative healing responses, even though all procedures were carried out by the same surgical team following a standard protocol.

## 5. Conclusions

In orthognathic surgery, this study showed that NemoFAB offers a dependable digital environment for predicting specific postoperative dentoskeletal changes. The virtual surgical plan and postoperative results consistently showed the highest degree of agreement, especially for parameters like overjet, overbite, maxillary incisor inclination, incisor exposure, and mandibular incisor projection with respect to the true vertical line that are mostly affected by sagittal and vertical jaw repositioning.

The conceptual role of virtual planning as a target configuration rather than a direct replication of the initial anatomical condition is reflected in the consistently lower agreement between preoperative measurements and virtual predictions. On the other hand, parameters that showed little variation and inherent biological stability, like maxillary height, B-point position, and pogonion projection, showed smaller predictive differences. Overall, these results show that NemoFAB’s predictive accuracy is parameter-dependent and mainly relates to skeletal and dentoalveolar variables that are directly impacted by surgical repositioning. As a result, it is not appropriate to interpret the current findings as a thorough three-dimensional validation of every anatomical component.

NemoFAB is a useful tool for increasing the predictability of sagittal and vertical treatment outcomes, facilitating interdisciplinary communication, and improving diagnostic precision within these specified limitations. It is anticipated that future advancements in artificial intelligence-driven landmark identification, soft-tissue modeling, and three-dimensional postoperative validation will improve the precision and practicality of virtual surgical planning systems.

## Figures and Tables

**Table 1:** Repeated Measures ANOVA (Pre vs. Post vs. Nemo).

Variable	Pre Mean (SD)	Post Mean (SD)	Nemo Mean (SD)	F-Statistic	*p*-Value
Overjet	5.28 (2.65)	3.30 (1.59)	3.50 (1.00)	13.66	0.00000820
Overbite	3.06 (2.17)	1.86 (1.29)	2.44 (1.15)	6.942	0.00168
Mx1 to Mx plane	57.15 (4.16)	60.71 (5.75)	61.11 (7.41)	16.474	0.00000108
Md1 to Md plane	73.84 (11.92)	72.96 (7.71)	72.45 (7.87)	0.699	0.500217
Mx1 exposure	3.93 (2.58)	2.34 (2.14)	2.93 (1.36)	12.066	0.0000272
Maxillary height	24.50 (4.39)	25.12 (4.32)	24.80 (7.78)	0.205	0.815322
Mx1 to TVL projection	−13.84 (2.81)	−12.54 (3.77)	−10.96 (6.10)	5.701	0.004886
Md1 to TVL projection	−8.16 (8.28)	−15.84 (4.21)	−15.31 (4.01)	31.419	0.000000000981
B-point to TVL projection	−5.62 (7.81)	−6.38 (5.37)	−7.56 (5.65)	2.527	0.086393
Pogonion to TVL projection	−3.06 (8.73)	−3.27 (5.94)	−3.54 (5.36)	0.147	0.863289
Mx occlusal plane to TVL	98.71 (5.29)	95.92 (4.29)	94.44 (4.88)	16.08	0.00000142

Preoperative, postoperative, and virtual surgical planning (Nemo) measurements were compared using repeated-measures ANOVA. A statistically significant difference between at least two time points is indicated by a significant *p*-value.

**Table 2:** Paired T test illustrates the variations between measurements of various parameters taken before and after surgery.

Variable	Mean Pre (SD)	Mean Post (SD)	Mean Difference	t-Statistic	Cohen’s d	*p*-Value
Overjet	5.28 (2.65)	3.30 (1.59)	1.981	3.816	0.603	0.000472
Overbite	3.06 (2.17)	1.86 (1.29)	1.202	3.149	0.498	0.003138
Mx1 to Mx occlusal plane	57.15 (4.16)	60.71 (5.75)	−3.56	−4.543	−0.718	0.0000523
Md1 to Md occlusal plane	73.84 (11.92)	72.96 (7.71)	0.883	0.637	0.101	0.527709
Mx1 exposure	3.93 (2.58)	2.34 (2.14)	1.586	3.955	0.625	0.000313
Maxillary height	24.50 (4.39)	25.12 (4.32)	−0.621	−1.229	−0.194	0.226619
Mx1 to TVL projection	−13.84 (2.81)	−12.54 (3.77)	−1.307	−1.911	−0.302	0.063440
Md1 to TVL projection	−8.16 (8.28)	−15.84 (4.21)	7.673	5.932	0.938	0.000000644
B-point to TVL projection	−5.62 (7.81)	−6.38 (5.37)	0.761	0.886	0.140	0.380879
Pogonion to TVL projection	−3.06 (8.73)	−3.27 (5.94)	0.217	0.222	0.035	0.825843
Mx occlusal plane to TVL	98.71 (5.29)	95.92 (4.29)	2.788	3.442	0.544	0.00139

Paired *t*-tests were used to evaluate differences between preoperative and postoperative measurements. The interpretation of Cohen’s d values was as follows: 0.2 = small effect, 0.5 = moderate effect, and ≥0.8 = large effect size.

**Table 3:** Comparison of Preoperative and Nemo Simulation Measurements Using Paired *t*-Tests.

Variable	Mean Pre (SD)	Mean Nemo (SD)	Mean Difference	t-Statistic	Cohen’s d	*p*-Value
Overjet	5.28 (2.65)	3.50 (1.00)	1.776	3.692	0.584	0.000680
Overbite	3.06 (2.17)	2.44 (1.15)	0.621	1.613	0.255	0.114796
Mx1 to Mx occlusal plane	57.15 (4.16)	61.11 (7.41)	−3.951	−4.082	−0.645	0.000214
Md1 to Md occlusal plane	73.84 (11.92)	72.45 (7.87)	1.392	0.919	0.145	0.363725
Mx1 exposure	3.93 (2.58)	2.93 (1.36)	1.000	2.795	0.442	0.008016
Maxillary height	24.50 (4.39)	24.80 (7.78)	−0.293	−0.246	−0.039	0.807233
Mx1 to TVL projection	−13.84 (2.81)	−10.96 (6.10)	−2.886	−3.062	−0.484	0.003969
Md1 to TVL projection	−8.16 (8.28)	−15.31 (4.01)	7.149	5.447	0.861	0.000003032
B-point to TVL projection	−5.62 (7.81)	−7.56 (5.65)	1.938	1.742	0.275	0.089475
Pogonion to TVL projection	−3.06 (8.73)	−3.54 (5.36)	0.482	0.418	0.066	0.677893
Mx occlusal plane to TVL	98.71 (5.29)	94.44 (4.88)	4.269	4.824	0.763	0.0000218

The differences between preoperative measurements and virtual surgical simulations were assessed using paired *t*-tests. The interpretation of Cohen’s d values was as follows: 0.2 = small effect, 0.5 = moderate effect, and ≥0.8 = large effect size.

**Table 4:** Bland–Altman Agreement Analysis Between Preoperative, Nemo, and Postoperative Measurements.

Comparison	Variable	Bias	SD (Diff)	LoA Low	LoA High
Pre vs. Nemo	Overjet	1.776	3.042	−4.186	7.737
Pre vs. Nemo	Overbite	0.621	2.434	−4.150	5.391
Pre vs. Nemo	Mx1 to Mx plane	−3.951	6.122	−15.951	8.048
Pre vs. Nemo	Md1 to Md plane	1.392	9.579	−17.383	20.167
Pre vs. Nemo	Mx1 exposure	1.000	2.263	−3.436	5.436
Pre vs. Nemo	Maxillary height	−0.293	7.550	−15.091	14.504
Pre vs. Nemo	Mx1 to TVL	−2.886	5.960	−14.566	8.795
Pre vs. Nemo	Md1 to TVL	7.149	8.301	−9.121	23.419
Pre vs. Nemo	B-point to TVL	1.938	7.038	−11.857	15.733
Pre vs. Nemo	Pogonion to TVL	0.482	7.288	−13.803	14.767
Pre vs. Nemo	Mx plane to TVL	4.269	5.598	−6.702	15.241
Pre vs. Post	Overjet	1.981	3.284	−4.455	8.418
Pre vs. Post	Overbite	1.202	2.413	−3.528	5.931
Pre vs. Post	Mx1 to Mx plane	−3.560	4.955	−13.271	6.152
Pre vs. Post	Md1 to Md plane	0.883	8.764	−16.295	18.061
Pre vs. Post	Mx1 exposure	1.586	2.536	−3.385	6.557
Pre vs. Post	Maxillary height	−0.621	3.196	−6.884	5.643
Pre vs. Post	Mx1 to TVL	−1.307	4.327	−9.788	7.174
Pre vs. Post	Md1 to TVL	7.673	8.181	−8.362	23.708
Pre vs. Post	B-point to TVL	0.761	5.429	−9.879	11.401
Pre vs. Post	Pogonion to TVL	0.217	6.188	−11.912	12.346
Pre vs. Post	Mx plane to TVL	2.788	5.122	−7.251	12.826
Nemo vs. Post	Overjet	0.206	0.904	−1.566	1.978
Nemo vs. Post	Overbite	0.581	0.857	−1.099	2.261
Nemo vs. Post	Mx1 to Mx plane	0.392	2.649	−4.800	5.584
Nemo vs. Post	Md1 to Md plane	−0.509	1.333	−3.121	2.103
Nemo vs. Post	Mx1 exposure	0.586	1.112	−1.594	2.766
Nemo vs. Post	Maxillary height	−0.327	6.771	−13.598	12.943
Nemo vs. Post	Mx1 to TVL	1.578	5.800	−9.789	12.946
Nemo vs. Post	Md1 to TVL	0.524	2.129	−3.650	4.697
Nemo vs. Post	B-point to TVL	−1.177	3.396	−7.834	5.479
Nemo vs. Post	Pogonion to TVL	−0.265	1.910	−4.010	3.479
Nemo vs. Post	Mx plane to TVL	−1.482	3.545	−8.431	5.467

The agreement between measurements was evaluated using Bland–Altman analysis. 95% limits of agreement and mean bias are presented. Higher concordance is indicated by narrower limits of agreement and smaller mean differences.

**Table 5:** Intraclass Correlation Coefficients (ICC) for Agreement Between Preoperative, Nemo, and Postoperative Measurements.

Variable	ICC(2,k) Global	Classification	ICC Pre–Nemo	Class	ICC Pre–Post	Class	ICC Nemo–Post	Class
Overjet	0.025	Poor	−0.112	Poor	-0.094	Poor	0.763	Good
Overbite	0.381	Poor	0.021	Poor	0.073	Poor	0.681	Moderate
Mx1 to Mx plane	0.816	Good	0.400	Poor	0.414	Poor	0.921	Excellent
Md1 to Md plane	0.864	Good	0.551	Moderate	0.622	Moderate	0.984	Excellent
Mx1 exposure	0.710	Moderate	0.359	Poor	0.352	Poor	0.770	Good
Maxillary height	0.694	Moderate	0.291	Poor	0.728	Moderate	0.427	Poor
Mx1 to TVL	0.483	Poor	0.182	Poor	0.144	Poor	0.334	Poor
Md1 to TVL	0.439	Poor	0.117	Poor	0.135	Poor	0.862	Excellent
B-point to TVL	0.830	Good	0.455	Poor	0.673	Moderate	0.795	Good
Pogonion to TVL	0.857	Good	0.499	Poor	0.662	Moderate	0.943	Excellent
Mx plane to TVL	0.686	Moderate	0.296	Poor	0.377	Poor	0.672	Moderate

To calculate intraclass correlation coefficients, a two-way random-effects model with absolute agreement was employed [ICC(2,k)]. The following ICC values were interpreted: 0.50 poor agreement, 0.50–0.75 moderate agreement, 0.75–0.90 good agreement, and >0.90 excellent agreement.

## Data Availability

All data regarding this manuscript can be requested from the corresponding author at talpos.serban@umft.ro and andra.stancioiu@umft.ro.
